# Correlation between adenoma detection rate and polyp detection rate at endoscopy in a non-screening population

**DOI:** 10.1038/s41598-020-58963-y

**Published:** 2020-02-10

**Authors:** B. Murphy, E. Myers, T. O’Shea, K. Feeley, B. Waldron

**Affiliations:** 1 Department of General Surgery, University Hospital Kerry, Tralee, Co. Kerry Ireland; 2 Institute of Technology Tralee, Tralee, Co. Kerry Ireland

**Keywords:** Colorectal cancer, Colonoscopy

## Abstract

It is understood that colorectal adenomas progress to colonic adenocarcinoma. Adenoma detection rate (ADR) at endoscopy has been used as a key performance indicator at endoscopy and is inversely associated with diagnosis of interval colorectal cancer. As most endoscopy reporting systems do not routinely incorporate histological assessment, ADR reporting is a cumbersome task. Polyp Detection Rate (PDR) has therefore been adopted as a surrogate marker for ADR. A prospectively maintained database of colonoscopies performed between July 2015 and July 2017 was analysed. This was cross referenced with a histological database. Statistical analysis was performed using IBM SPSS, version 24. Inferential procedures employed included the Pearson’s correlation coefficient (*r*) and Binomial logistic regression. Of 2964 procedures performed by 8 endoscopists, overall PDR was 27% and ADR was 19%. The PDR, ADR, adenoma to polyp detection rate quotient (APDRQ) and estimated ADR (PDR x APDRQ group average = 0.72) was calculated for each individual. There was a strong positive linear correlation between PDR and ADR,*r*(8) = 0.734, *p* = 0.038 and between PDR and estimated ADR, *r*(8) = 0.998, *p < *0.001. Adenoma detection rate strongly correlated with estimated ADR, *r*(8) = 0.720, *p* = 0.044. With the exclusion of a moderate outlier, these correlations increased in both strength and significance. There was a stronger correlation between PDR and ADR,*r*(7) = 0.921, *p* = 0.003 and between ADR and estimated ADR, *r*(7) = 0.928, *p* = 0.003.

## Introduction

Colorectal cancer (CRC) is the second most common cancer diagnosed in Ireland. It is the third leading cause of death in women and the second leading cause of cancer death in men^1^. Adenocarcinoma accounts histologically for > 95% of malignant tumours and it is now widely accepted that the majority of cases appear to arise from the progression of adenomatous polyps to invasive malignancy^2,3^. The adenoma to carcinoma sequence describes the progression of normal tissue to dysplastic tissue and ultimately to carcinoma^2^. This has enhanced genetic knowledge of colorectal cancers as well as highlighted the importance of polyp surveillance and removal at endoscopy. The adenoma detection rate (i.e. proportion of procedures where one or more adenomas are detected) at colonoscopy has been shown to be inversely associated with interval cancer development, advanced–stage interval cancer and fatal interval cancer^4,5^. High quality endoscopy and adequate adenoma detection rate (ADR) are hence paramount in the prevention and treatment of CRC.

Key Performance Indicators and Quality Assurance Standards for endoscopy are the minimum standards to which an endoscopist should perform in certain crucial aspects of the procedure e.g. unadjusted caecal intubation rate > 90%. The British Society of Gastroenterology (BSG), the UK Joint Advisory Group on GI Endoscopy (JAG), and the Association of Coloproctology of Great Britain and Ireland (ACPGBI) have established a working group to develop key performance indicators for the delivery of colonoscopy as a measure of quality assurance in the United Kingdom and Ireland^5^. This working group has suggested a minimum standard for adenoma detection rate at 15% with an aspirational ADR of 20%^5^. The main limitation in utilising ADR as a KPI however is this requires histological examination of the polyp to confirm its adenomatous aetiology. Most endoscopy reporting systems require report completion at the time of the procedure with no standard interfacing between the reporting software and the histological outcome. Endoscopists thus frequently report *polyp* detection as opposed to *adenoma* detection at the time of colonoscopy. Once the histology results are available, the only means of linking these to the endoscopy report is usually by a time-consuming “hand audit”. Although a seemingly subtle difference between the PDR and ADR, this can have major clinical implications.

Among the several potential pitfalls of using PDR routinely is that not all reported polyps removed endoscopically will be adenomatous, and the proportion of neoplastic (adenomatous) to non-neoplastic polyps (e.g. hyperplastic polyps) which carry virtually no malignant potential may vary significantly among institutions. Furthermore, the concept of *identified* polyps does not infer that these polyps are universally *retrieved* which may result in further discrepancy between PDR and ADR.

The UK and Ireland working group propose that PDR may be used as a marker of ADR once the validity of same is confirmed for the unit. This is a somewhat ambiguous comment and it remains unclear how best one should do this. Previous studies have demonstrated that by using a conversion factor, an endoscopy unit can accurately estimate ADR using PDR. This encompasses calculation of the average adenoma to polyp detection rate quotient (APDRQ) for a diverse group of endoscopists. This quotient may be expressed as a fraction of the PDR to give an estimated Adenoma Detection Rate (eADR). This has been shown to correlate at a significant level with actual ADR^6–8^. Further studies have demonstrated a linear relationship between PDR and ADR^9^ to identify a PDR that will correlate with the minimum standard of ADR. It has also been shown however that this relationship is not constant when comparing the right colon to left colon^10–12^. This is due to the increased excision of smaller non-neoplastic polyps (e.g. hyperplastic polyps) in the left colon which increases the PDR but not the ADR^10^. This casts further criticism on the validity of PDR alone as a KPI, suggesting that a constant linear relationship between the two cannot be attributed with confidence.

The goal of this study therefore was to investigate the validity of PDR as a surrogate marker for ADR in an Irish hospital setting.

## Materials and Methods

All complete colonoscopies were analysed retrospectively between July 2015 and July 2017. All patients over 18 years of age were included. A complete colonoscopy was considered as that to the caecum/terminal ileum or in the case of previous ileocaecal resection, ileocolic or jejenocolic anastomosis. The polyp detection rate was analysed using the standard endoscopy reporting software at our institution. Similar to most endoscopy reporting systems, the performing endoscopist recorded data at the time of the procedure to produce an immediate report. PDR was taken to mean the proportion of colonoscopies where > / = 1 polyp were identified. Patient demographics, procedure/withdrawal times and sedation dosages were also obtained using this system. Screening colonoscopies were excluded as the histology from these procedures is routinely analysed at another institution.

The adenoma detection rate (i.e. procedures in which > / = 1 histologically-confirmed adenoma was detected) was analysed using a prospectively maintained database in the department of histopathology at our institution. This was performed for each patient in whom polyps were detected and removed by means of matching the systems via unique patient hospital numbers. Using these databases, we were able to calculate the PDR, ADR, adenoma to polyp detection rate quotient (APDRQ) and estimated ADR (PDR x APDRQ group average = 0.72) for our unit.

Statistical analysis was performed using IBM SPSS, version 24. Inferential procedures employed included the Pearson’s product-moment correlation coefficient (*r*) and Binomial logistic regression.

Informed consent was taken from all patients undergoing colonoscopy for their anonymised data including histology results to be utilised. Ethical approval was sought and granted by our institutional ethics board.

## Results

Included for analysis were 3,274 procedures on 3,079 patients over a 2-year period from July 2015 to July 2017. The discrepancy between patient numbers and procedure numbers is because some patients had more than one colonoscopy during this time. All procedures were performed by 20 departmental endoscopists of varying experience and seniority. One or more polyps were detected in 790 patients, giving an overall PDR of 26.7%. As anticipated, a higher proportion of polyps were detected with increasing age (Table 1).

**Table 1:** Proportion of polyps detected within each age category (n = 3079 Patients).

		Polyps Detected		
		No	Yes	Total
Age	18–29	96.4%	3.6%	100.0% (112)
	30–39	89.7%	10.3%	100.0% (319)
	40–49	79.8%	20.2%	100.0% (471)
	50–59	76.3%	23.7%	100.0% (611)
	60–69	71.4%	28.6%	100.0% (683)
	70–79	63.0%	37.0%	100.0% (611)
	80–89	66.0%	34.0%	100.0% (253)
	90 and above	68.4%	31.6%	100.0% (19)
Total		74.3%	25.7%	100.0% (3079)

One or more adenomas were detected by the histopathology department in 584 patients, confirming an overall adenoma detection rate of 19.6%. Of these, 17.7% were colonic adenomas, 0.7% rectal adenomas and 1.2% sessile serrated adenomas. 6.4% of patients < 50 had one or more adenomas compared with 25.8% in the cohort > 50. The proportion of adenomas in patients according to age group is outlined in Table 2.

**Table 2:** Proportion of adenomas assigned within each age category (n = 3079 Patients).

		Adenomas Assigned		Total
		No	Yes	
Age	18–29	98.2%	1.8%	100.0% (112)
	30–39	94.0%	6.0%	100.0% (319)
	40–49	88.5%	11.5%	100.0% (471)
	50–59	84.0%	16.0%	100.0% (611)
	60–69	78.0%	22.0%	100.0% (683)
	70–79	70.7%	29.3%	100.0% (611)
	80–89	70.0%	30.0%	100.0% (253)
	90 and above	68.4%	31.6%	100.0% (19)
Total		81.0%	19.0%	100.0% (3079)

On further examination of the database, it was noted that 8 staff members accounted for > 90% of procedures performed in the department. These 8 endoscopists had performed between 189 and 648 procedures during the 2-year period and comprised 2 consultant surgeons, 1 consultant gastroenterologist, 1 advanced nurse practitioner in endoscopy and 4 senior surgical registrars. To obtain a more accurate representation of what the estimated departmental ADR was, we reanalysed the data looking at these 8 endoscopists alone. This adjustment was also in keeping with previous studies where departments included endoscopists performing more than 200 colonoscopies per year^6^. Ours is a smaller institution where this volume of numbers was not feasible to conduct a representative study in our department, but our procedure numbers are nonetheless reflective of endoscopic practices within Ireland.

On analysis of the 2964 procedures performed by the 8 endoscopists with the highest procedure numbers, the PDR and ADR remained relatively unchanged at 27% and 19%, respectively. Each individual’s overall performance was analysed and the PDR, ADR, adenoma to polyp detection rate quotient (APDRQ) and estimated ADR (PDR x APDRQ group average = 0.72) calculated (Tables 3 and 4).

**Table 3:** PDR for 8 Individuals.

Based on 2964 procedures performed by 8 Individuals				
	Procedures	Zero PD	≥ 1 PD	PDR
Endoscopist 1	288	219	69	0.24
Endoscopist 2	648	431	217	0.33
Endoscopist 3	225	153	72	0.32
Endoscopist 4	283	229	54	0.19
Endoscopist 5	189	129	60	0.32
Endoscopist 6	547	445	102	0.19
Endoscopist 7	298	212	86	0.29
Endoscopist 8	486	357	129	0.27
Total	2964	2175	789	0.27

**Table 4:** ADR for 8 Individuals.

Based on 2964 procedures performed by 8 Individuals				
	Procedures	Zero AD	≥ 1 AD	ADR
Endoscopist 1	288	220	68	0.24
Endoscopist 2	648	501	147	0.23
Endoscopist 3	225	182	43	0.19
Endoscopist 4	283	248	35	0.12
Endoscopist 5	189	144	45	0.24
Endoscopist 6	547	470	77	0.14
Endoscopist 7	298	235	63	0.21
Endoscopist 8	486	402	84	0.17
Total	2964	2402	562	0.19

Pearson’s product-moment correlation was used to evaluate the strength of the relationship between a) Actual ADR and Estimated ADR, b) PDR and Actual ADR and c) PDR and Estimated ADR. This exercise was run on two occasions; the first with the 8 endoscopists and the second with 7 endoscopists excluding one moderate outlier.

There was a strong positive linear correlation between PDR and ADR, *r*(8) = 0.734, *p* = 0.038 and between PDR and estimated ADR, *r*(8) = 0.998, *p < *0.001. Similarly, Adenoma detection rate strongly correlated with estimated ADR, *r*(8) = 0.720, *p* = 0.044. In fact, with the exclusion of a moderate outlier, these correlations increased in both strength and significance. There was an even stronger correlation between PDR and ADR *r*(7) = 0.921, *p* = 0.003 and between ADR and estimated ADR, *r*(7) = 0.928, *p* = 0.003 (Tables 5 and 6).

**Table 5:** Correlations with 8 endoscopists.

PDR			ADR	Est_ADR
PDR	Pearson Correlation	1	0.734^*^	0.998^**^
	Sig. (2-tailed)		0.038	0.000
	N	8	8	8
ADR	Pearson Correlation	0.734^*^	1	0.720^*^
	Sig. (2-tailed)	0.038		0.044
	N	8	8	8
Est_ADR	Pearson Correlation	0.998^**^	0.720^*^	1
	Sig. (2-tailed)	0.000	0.044	
	N	8	8	8

*Correlation is significant at the 0.05 level (2-tailed).

**Correlation is significant at the 0.01 level (2-tailed).

**Table 6:** Correlations with 7 endoscopists with the exclusion of one outlier.

PDR			ADR	Est_ADR
PDR	Pearson Correlation	1	0.921^**^	0.998^**^
	Sig. (2-tailed)		0.003	0.000
	N	7	7	7
ADR	Pearson Correlation	0.921^**^	1	0.928^**^
	Sig. (2-tailed)	0.003		0.003
	N	7	7	7
Est_ADR	Pearson Correlation	0.998^**^	0.928^**^	1
	Sig. (2-tailed)	0.000	0.003	
	N	7	7	7

**Correlation is significant at the 0.01 level (2-tailed).

## Discussion

PDR has been proposed as an acceptable surrogate marker for ADR as a key performance indicator in endoscopy^5^. As previously discussed, this has been adopted due to the difficult task of routinely matching histological results to endoscopy reporting systems. It has been suggested that a PDR of 20% is the minimum standard to which an endoscopist should perform, as adapted from a proposed minimum adenoma detection rate of 15%. While the specialist bodies BGS, JAG and ACPGBI support the use of PDR as an estimate of ADR, this is based on the prerequisite that PDR be validated as a marker on an individual unit level^5^.

Previous research demonstrated that by using a conversion factor, it was possible to accurately estimate a departmental ADR using the PDR^6–8^. This significantly correlated with the actual adenoma detection rate, suggesting that in the absence of the true adenoma detection rate, this can be used as a reliable indicator. Similarly, in our study, we demonstrated that the estimated polyp detection rate as determined by the group average significantly correlated with individual adenoma detection rates. As is also evident from our study however, one group outlier may affect the ADPRQ and thus potentially weaken the validity of its usage. This emphasises the importance of continuous audit of practice within a unit and regular reporting of unit data and KPI targets.

Regarding the 8 endoscopists accounting for the majority of colonoscopies performed during this study timeframe, the ADR significantly correlated with the PDR (*p* = 0.038). The calculations were performed again with the exclusion of the moderate outlier. These findings, with the exclusion of this outlier revealed both increased strength and significance, *r*(7) = 0.921, *p* = 0.003. (Tables 5 and 6). The outlying endoscopist was such as they had no discrepancy between their individual ADR and PDR (24%) giving and ADPRQ of 1; this infers that every polyp excised was in fact adenomatous. Given the retrospective nature of our study, this finding is likely due to erroneous data recording at the time of procedure rather than truly reflective of endoscopists practice but this could not be formally assessed.

This paper has limitations, some of which have previously been addressed. As mentioned, it is retrospective and therefore subject to inaccuracies in data reporting and collection. The practicing endoscopists in the studied endoscopy unit vary in procedure numbers and experience which leads to a heterogeneity of the group. This nevertheless is likely reflective of actual practice in many endoscopy units throughout training hospitals and is an important factor to note when assessing an endoscopy unit’s data and KPI reporting. Furthermore, the eADR as determined by the group average ADR correlated significantly with the individual ADR for all endoscopists, suggesting that experience was not a major factor influencing ADR in our unit. Again, further studies in this department would be needed to assess this.

Endoscopist experience and correlation of same with ADR or PDR was not looked at in this study. Previous papers addressing this question have produced conflicting results with some demonstrating no difference between trainees and consultants^12^ and others showing a significant correlation between ADR and number of years’ endoscopy experience^13^. The discrepancies between endoscopists in our study were nevertheless highly interesting. While some had significantly higher polyp detection rates compared to that of adenoma detection rates, others had nearly equal rates. One individual had an identical adenoma and polyp detection rate of 24% and was thus excluded as a moderate outlier during statistical analysis. This may reflect different levels of experience i.e. this endoscopist may more easily recognise polypoid tissue without malignant potential such as hyperplastic polyps and therefore not remove them endoscopically. This however is not the standard practice in our unit where all polypoid tissue is removed where feasible. We hypothesise therefore that these figures have resulted from erroneous data recording. Interestingly, higher polyp detection rates among our endoscopists did not in fact result in higher adenoma detection rates as compared with their peers and in fact, the moderate outlier had the highest ADR (24%) along with one other endoscopist with the same rate.

In keeping with this, a pertinent question with the increasing pressure on endoscopists to meet KPI standards is whether more non-neoplastic polyps (i.e. hyperplastic polyps or normal tissue) are being removed to increase the PDR of an individual or unit. One can argue however that without a high level of training or experience in adenoma recognition, it is prudent to remove all polyps suspected of adenomatous aetiology minimising the risk of dysplastic tissue being left behind inadvertently. Improved education for trainees regarding recognition of adenomas via morphological pit patterns^14^ and technology availability such as chromoendoscopy and narrow band imaging^15,16^ may result in improved adenoma detection rates with perhaps lower rates of non-neoplastic polyp excision.

## Conclusions

In this study, we have demonstrated that PDR may be used as a reliable indicator of ADR within an endoscopy unit. Adopting this practice however is not without potential flaw. Firstly identified polyps as is measured in PDR does not infer that these polyps are retrieved which may result in a discrepancy. Furthermore, a constant linear relationship between PDR and ADR is not demonstrable throughout the colon while comparing the right to the left side^10,11^. In keeping with previous research, we have shown that an estimated ADR by means of a conversion factor significantly correlated with actual ADR and can be considered as a feasible alternative to PDR reporting.

Ideally, integration of true ADR calculation in all endoscopy units should replace the surrogate marker of PDR. A means of doing this efficiently and in a standardised fashion however has yet to be developed to the authors’ knowledge. We conclude therefore that until endoscopy reporting software is automatically interfaced with histological outcomes, PDR or an estimated ADR by means of a conversion factor may be used as a reliable indicator of quality endoscopy once validated for the unit in question.
